# Clinical Utility of Coil in Plug Method (CIP) for Internal Iliac Artery Embolization during Endovascular Aortic Aneurysm Repair

**DOI:** 10.3400/avd.oa.20-00034

**Published:** 2020-09-25

**Authors:** Akiyuki Kotoku, Yukihisa Ogawa, Kiyoshi Chiba, Takaaki Maruhashi, Hidefumi Mimura, Takeshi Miyairi, Hiroshi Nishimaki

**Affiliations:** 1 Department of Radiology, St. Marianna University, School of Medicine; 2 Department of Cardiovascular Surgery, St. Marianna University, School of Medicine; 3 Department of Emergency and Critical Care Medicine, Kitasato University School of Medicine

**Keywords:** coil in plug, internal iliac artery, embolization, EVAR

## Abstract

**Objective**: To evaluate the clinical utility of the coil in plug (CIP) method in internal iliac artery (IIA) embolization during endovascular aortic aneurysm repair (EVAR) compared to conventional coil embolization (CCE).

**Material and Methods**: From July to December 2018, 10 patients who underwent IIA embolization during EVAR were divided into CIP (n=5) and CCE (n=5) groups. In the CIP technique, the AVP-1 with a size more than 30%–50% of that of the embolized IIA diameter was used. The AVP-1 was deployed in the IIA. Before detachment of the AVP-1, a 2.2-F micro catheter was inserted through the 6-F delivery guiding sheath, and entered the plug. The AVP-1 was then packed with hydrogel micro coils.

We compared number of coils used, embolization length, embolization time, volume embolization ratio, and embolic material cost between the groups.

**Results**: The CIP method achieved shorter embolization length with fewer coils used compared to CCE. The CIP method decreased the cost of total embolic materials.

**Conclusion**: The CIP method can achieve shorter embolization length with fewer coils used compared to CCE.

## Introduction

Endovascular aortic aneurysm repair (EVAR) needs internal iliac artery (IIA) embolization when the peripheral sealing zone is short or when a common iliac artery aneurysm exists.

Standard occlusion of the IIA has generally been performed by conventional coil embolization (CCE). However, CCE often needs multiple coils, which incur a high cost,^1)^ and has the risk of coil migration to the external iliac artery when embolizing the proximal IIA.

The Amplatzer Vascular Plug series (Abbott Vascular, Redwood City, CA, USA) is an ideal occlusion device for IIA embolization. The AVP-1 has a short length that can reduce the risk of buttock claudication or other ischemia because of preserving branches of the IIA, but has a potential risk of incomplete embolization or recanalization, especially in a high flow situation recanalization.^2,3)^ Koganemaru et al.^4,5)^ and Katada et al.^3)^ reported a method for putting coils into AVP-1 called coil in plug (CIP) to solve this issue. They achieved complete short-segment embolization in high flow vessels.

This study evaluated the clinical utility of CIP in IIA embolization during EVAR compared to CCE.

## Materials and Methods

This retrospective study was performed at the St. Marianna University School of Medicine. The requirement for obtaining approval of the ethics committee was waived for this procedure because all devices used were approved for endovascular treatment. Written informed consent was obtained from all patients.

From July to December 2018, 10 patients who underwent IIA embolization during EVAR were divided into CIP (n=5) and CCE (n=5) groups. The CCE was performed before introducing the CIP technique. IIA diameter in the CCE group was matched for the CIP group.

All patients had both abdominal aortic aneurysm (AAA) and common iliac artery aneurysm (CIAA), and they were asymptomatic. Patient summaries are shown in **Table 1**.

**Table 1:** Table 1 Patient demographics and lesion characteristics

	CIP (n=5)	CCE (n=5)	P value
Age	78.4±14.2	77.0±3.5	0.82
Male (%)	4 (80)	4 (80)	1
Diabetes mellitus	1	1	1
Hypertension	3	5	0.44
CKD (>stage 3; eGFR <60)	4	1	0.20
Dyslipidemia	4	2	0.52
COPD	2	1	0.99
Cardiovascular disease	3	4	0.99
Anti-coagulant/platelet	3	3	1
Mean IIA diameter (mm)	10.2±4.0	10.2±2.7	0.78
Mean AAA diameter (mm)	46.2±17.3	52.2±19.4	0.61
Mean CIAA diameter (mm)	28.0±7.3	39.4±17.2	0.32

CKD: chronic kidney disease; COPD: chronic obstructive pulmonary disease; IIA: internal iliac artery; AAA: abdominal aortic aneurysm; CIAA: common iliac artery aneurysm; CIP: coil in plug; CCE: conventional coil embolization

All procedures were performed under general anesthesia and heparinization (activated coagulation time >200 s).

### CIP Procedure (Fig. 1)

**Figure 1:**
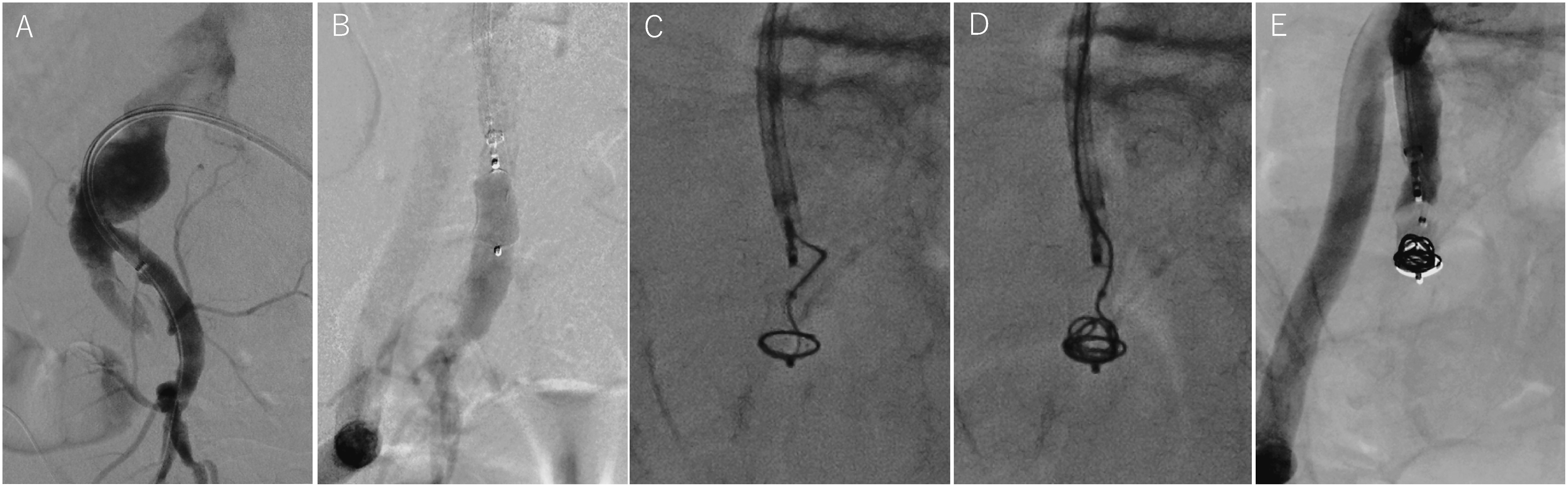
Fig. 1 Coil in plug technique. (**A**) All procedures were performed via a contralateral femoral artery approach with a long 6-F guiding sheath. (**B**) AVP-1 (10%–20% oversized to internal iliac artery (IIA) diameter) was deployed in the IIA. (**C**) Before detachment of the AVP-1, a 2.2-F micro catheter was inserted through the 6-F delivery guiding sheath into the AVP-1 using a 0.014” micro-wire. (**D**) Put hydrogel micro coils into the AVP-1 until complete embolization. The size of the first coil was equal to the embolized IIA, followed by a smaller size. (**E**) Completion angiogram after the procedure shows complete embolization.

All embolization procedures were performed via a contralateral femoral artery approach with a long 6-F guiding sheath (Parent Plus; Medikit, Tokyo, Japan)(**Fig. 1A**).

The AVP-1 with a size more than 30%–50% of that of the embolized IIA diameter was used.

The AVP-1 was deployed in the IIA (**Fig. 1B**). Before detachment of the AVP-1, a 2.2-F micro catheter (Coiling Support; HI-LEX corporation, Takarazuka, Japan) was inserted through the 6-F delivery guiding sheath into the AVP-1 using a 0.016-inch guide-wire (AQUA VIII, ASAHI intecc, Aichi, Japan)(**Fig. 1C**). The AVP was packed with hydrogel micro coils (AZUR18: Terumo, Tokyo, Japan) until confirming complete embolization from the delivery sheath (**Figs. 1D** and **1E**).

### CCE Procedure

Embolization of IIA was performed via a contralateral or ipsilateral femoral arterial approach. An 8-F introducer sheath was placed from both femoral arteries. The IIA was selected by a 5-F catheter (cobra-shaped or shephard-hook-shaped) with or without coaxial system consisting of a 2.7-F microcatheter (Coiling Support EX; HI-LEX corporation) and 0.016-inch micro-wire (AQUA VIII), and was embolized with detachable coils. We used various coils; Hydrocoils (AZUR35, AZUR CX35, AZUR18 or AZUR CX18: Terumo, Tokyo, Japan), or other detachable coils such as Penumbra (Penumbra Inc., Alameda, CA, USA), and Micrasframe or Deltafill (Cerenovus, Tokyo, Japan).

### Endpoints

The primary endpoint was technical success defined as complete embolization of the IIA with accurate deployment without any migration of the embolic materials.

Secondary endpoints include number of coils used, embolization length, embolization time, volume embolization ratio (VER), passing time, recanalization, buttock claudication, and embolic material cost that were compared between the groups.

Embolization time was duration from time of first IIA angiography to complete embolization.

The VER was defined as [π×(coil radius)^2^×(coil length)/ π×(vessel diameter)^2^×embolization length/4], which was calculated by the AngioCalc app. The embolization length was measured on the postoperative computed tomography.

Passing time was defined as the time from the deployment of AVP-1 to insertion of the microcatheter.

Statistical analysis was performed with SAS software (version 9.4; SAS Institute, Inc., Cary, NC, USA), and variables were compared between the groups using the χ^2^ or Fisher exact tests with p<.05 taken to indicate a significant contribution.

## Results

Technical success was achieved in four in the CIP group (80%), and five in the CCE group (100%).

One case had coil migration during the finishing process though it was recovered by pushing into the plug.

In one failure case that did not get the microcatheter into the plug due to aneurysmal space proximally that made it difficult to cannulate because of catheter instability, coil embolization of aneurysmal space was needed.

With complete embolization being achieved without any complication in both groups, the mean follow-up period was 9.6 months in the CIP group (range 2–14 months), and 11 months in the CCE group (range 4–16 months).

With there being neither recanalization nor buttock claudication in the CIP group, the CIP method achieved shorter embolization length with a fewer number of coils used, and decreased the cost of total embolic materials though it did not reach statistical significance. Mean passing time was 7.7 min in the CIP group. There were no significant differences of VER and embolization time between the groups.

The clinical results are shown in **Table 2**.

**Table 2:** Table 2 Clinical results

	CIP (n=5)	CCE (n=5)	P value
Technical success (%)	4 (80)	5 (100)	0.99
Number of coils used	3.8±1.2	8.8±3.9	0.016
Embolization length (mm)	19±3.8	38±6.1	0.026
VER (%)	24±18.6	19±6.6	0.66
Embolization time (min)	31±18.9	36±11.6	0.63
Passing time (min)*	7.7±4.5	N/A	N/A
Recanalization	0	0	1
Buttock claudication	0	1	0.99
Embolic material cost (¥)	705,000±287,813	1,185,000±405,729	0.12
Follow up period (month)	9.4±5.2	9.6±4.0	0.95

VER: volume embolization ratio; CIP: coil in plug; CCE: conventional coil embolization. *passing time: the time from deployment of AVP-1 to insertion of the microcatheter

## Discussion

The CIP method can achieve shorter embolization length with fewer coils used compared to CCE. Kritpracha et al.^6)^ recommended that IIA embolization should be performed as proximal as possible to prevent intervene pelvic collateral circulation. Finding a significantly higher incidence of pelvic ischemia (75%) in the distal embolization group compared to the 13% in the proximal embolization group, the proximal IIA embolization was compared with distal IIA embolization.

The AVP-1 has a potential risk of nonocclusion during the embolization procedure or recanalization at a later time, as it provides rapid occlusion with precise positioning in short landing zones, but its embolic effects can depend on vessel diameter, blood flow, clotting ability, and the size of the plug. An AVP-2 has a higher embolic effect, but its longer length can cause unplanned branch embolization.^7)^ Tuite et al.^8)^ reported that recanalization rate of the AVP-1 alone was 61% in high-flow situation. Although we needed to add coils proximally when the AVP-1 had incomplete embolization, the embolization length had to be extended, although this CIP method can make the AVP-1 embolic effect increase without extension of the embolization length.

Because the embolic effect of the plug did not affect VER, and the endpoint of putting coils into the plug was complete embolization, not apparent density, in the present study, VER in the CIP group was not significantly higher than that in the CCE group, despite the shorter embolization length.

We recommend making space for proximal embolization if the situation is complicated by there being tortuosity or aneurysmal change of the IIA, as it may not allow the microcatheter to enter the plug, which can result in technical failure. With this technique having potential risk of coil migration during the finishing process, even when the microcatheter is in the plug, we need to conduct modified techniques that definitely enable cannulation into the plug.

This short-segment embolization technique can be applied to other pathologies such as the left subclavian artery embolization during thoracic EVAR, pulmonary arteriovenous malformation, other arteriovenous fistula, or blood alternation before Appleby operation.

This study has several limitations. It was a retrospective study that involved a small number of patients. There is also selection bias in the CCE group since we matched for the IIA diameter. In addition, the follow-up period was short at less than one year, and the long-term outcomes are unknown.

## Conclusion

Compared to CCE, the CIP method can achieve shorter embolization length with fewer coils used. We need to conduct modified techniques enabling cannulation into the plug regardless of the situation.
